# Impact of lymphocyte differential count > 15% in BALF on the mortality of patients with acute exacerbation of chronic fibrosing idiopathic interstitial pneumonia

**DOI:** 10.1186/s12890-017-0412-8

**Published:** 2017-04-20

**Authors:** Reoto Takei, Machiko Arita, Shogo Kumagai, Yuhei Ito, Maki Noyama, Fumiaki Tokioka, Tadashi Ishida

**Affiliations:** Department of Respiratory Medicine, Kurashiki Central Hospital, 1-1-1 Miwa, Kurashiki, Okayama 710-0052 Japan

**Keywords:** Interstitial Lung Disease, Acute exacerbation, Lymphocyte differential count in BALF

## Abstract

**Background:**

Chronic fibrosing idiopathic interstitial pneumonia (CFIIP) has a potential risk of acute exacerbation (AE). However, the usefulness of cellular analysis of bronchoalveolar lavage fluid (BALF) has never been evaluated. This study aimed to evaluate the impact of the lymphocyte differential count > 15% in BALF on the mortality of patients with AE of CFIIP.

**Methods:**

We retrospectively analysed 37 patients with AE of CFIIP who underwent BAL on admission. Patients were divided into two groups: one group consisting of those with a lymphocyte differential count > 15% and the other consisting of those with a lymphocyte differential count ≤ 15%. We compared the 90-day mortality between the two groups as the primary outcome, using the two-tailed log-rank test.

**Results:**

The median follow-up duration was 6.9 months. Twenty-four patients had a lymphocyte differential count > 15%. The 90-day mortality was significantly higher in the group with a lymphocyte differential count ≤ 15% than in the group with a lymphocyte differential count > 15% (long rank test, *p* = 0.003). In the multivariate analysis a lymphocyte differential count > 15% was shown to be an independent favourable prognostic factor for 90-day mortality (HR: 0.125; 95% CI: 0.0247–0.589; *p* = 0.009).

**Conclusions:**

A lymphocyte differential count > 15% in BALF may be associated with favourable outcomes in patients with AE of CFIIP.

**Electronic supplementary material:**

The online version of this article (doi:10.1186/s12890-017-0412-8) contains supplementary material, which is available to authorized users.

## Background

Acute exacerbation (AE) in patients with idiopathic pulmonary fibrosis (IPF), characterised by clinically progressive respiratory failure and worsening pulmonary fibrosis, was first reported in 1993 and has been recognised as a relatively common and highly critical event in the clinical course of IPF [1, 2]. AE has also been described in other cases of fibrosing interstitial pneumonia [3–6], suggesting the potential risk of AE in chronic fibrosing idiopathic interstitial pneumonia (CFIIP).

The diagnostic consensus criteria for AE were suggested by Collard et al. in 2007 [7] and include a lack of evidence of pulmonary infection by endotracheal aspirate or bronchoalveolar lavage (BAL). However, there have been no studies of the specificity of the cellular analysis of BAL fluid (BALF) and appropriate treatment for AE has not been established, and so the mortality rate of AE remains high [8].

Although corticosteroids were weakly recommended in the American Thoracic Society/European Respiratory Society/Japanese Respiratory Society/Latin American Thoracic Association (ATS/ERS/JRS/ALAT) guidelines for patients with AE of IPF [9], no controlled trials on the effectiveness of corticosteroids have been conducted. According to the ATS/ERS guidelines, BAL cell differential counts > 15% lymphocytes represent a lymphocytic cellular pattern such as cryptogenic organizing pneumonia [10], which has been shown to have a good recovery with corticosteroid treatment [11]. So patients with BAL cell differential counts > 15% lymphocytes may be good responders to corticosteroids. However, the effect of corticosteroids and other immunosuppressants in AE of CFIIP with > 15% lymphocytes in BALF has never been evaluated.

In this study, we retrospectively analysed patients with AE of CFIIP who underwent bronchoscopy with BAL in order to evaluate the impact of the lymphocyte differential count > 15% in BALF on outcomes.

## Methods

### Study design

Patients with AE of CFIIP who were admitted to our department and underwent BAL examination between August 2006 and February 2015 were retrospectively studied. This study protocol was approved by the ethics committee of Kurashiki Central Hospital in accordance with the Declaration of Helsinki (approved number 1972). The categorization of major idiopathic interstitial pneumonias in ATS/ERS guidelines was used to define CFIIP [11].

### Criteria for AE

AE was defined using the following features based on the defined criteria [7]: (1) previous or concurrent diagnosis of CFIIP, (2) acute worsening or development of dyspnea within 1 month duration, (3) computed tomography with new bilateral ground-glass opacity and/or consolidation superimposed on a background pattern, (4) deterioration not fully explained by cardiac failure, fluid overload, or an identifiable cause of acute lung injury. A significant decline in oxygenation was confirmed by previous PaO_2_ or SpO_2_ in every patient. Drugs such as anticancer agents, herbal medicines, health foods, and other suspicious drugs were considered as the cause of acute lung injury when they were used within 30 days before admission, and these patients were excluded.

### BAL procedures

BAL was performed with a fiber-optic bronchoscope in a wedge position within the selected bronchopulmonary segment [10, 12]. Sterile saline (0.9% NaCl) at room temperature was instilled through the bronchoscope. The total instilled volume was 100 or 150 ml and was divided into two or three 50-ml aliquots. After instillation of each aliquot, the instilled sterile saline was retrieved using negative suction pressure adjusted to avoid visible airway collapse. The recovered BALF was separated from the cellular components by centrifugation (1 min, 1300 rpm). Preparations of cell suspensions were made in a cytocentrifuge (Shandon, Woburn, MASS). Cytospin slides of BALF cells were stained with May-Grünwald-Giemsa for cell differentiation.

### Clinical characteristics and laboratory findings

The patient’s clinical characteristics were retrieved from the available clinical record. A respiratory function test was performed within 1 year before AE. Two pulmonologists, who had no knowledge of any prior clinicopathological reports relevant to these cases, reviewed the computed tomography and classified the patients into two groups according to the ATS/ERS criteria as those with usual interstitial pneumonia (UIP) and those with not UIP [13].

### Statistical analysis

We divided the patients with AE of CFIIP into two groups; one group had a lymphocyte differential count > 15%, and the other group had a lymphocyte differential count ≤ 15%. We set 90-day mortality as the primary outcome and overall survival (OS) as the secondary outcomes. The 90-day mortality was measured at 90 days after the BAL procedures. The OS was also measured from the date of the BAL procedure until the date of death from any cause or until the date on which the patient was last known to be alive. The cause of death was regarded as AE when the patient died within 90 days from the date of performing BAL. We estimated the 90-day mortality and OS using Kaplan-Meier analysis [14]. Differences between survival curves were tested for statistical significance using the two-tailed log-rank test. Univariate and multivariate prognostic analyses were performed for 90-day mortality using logistic regression model. For multivariate analysis, a stepwise backward procedure to derive a final model of the variables that had a significant independent relationship with survival was employed. All variables with a *P* value of less than 0.10 in the univariate analyses were entered into the multivariate analysis. Categorical variables were compared using the Fisher test, and continuous variables were compared using the *t*-test or the Mann-Whitney *U* test. All statistical analyses were performed using R (The R Foundation for Statistical Computing V.3.0.3). Statistical significance was defined as a *p* value of <0.05. Data were expressed as the median (interquartile range) or mean ± standard deviation.

## Results

### Patient characteristics

A total of 37 patients met the study inclusion criteria. The median age at the time of BAL was 75 years (range: 72–78 years) and the median follow-up duration was 6.9 months (range: 1.7–19.1 months). Twenty-four patients showed lymphocyte differential counts > 15%. The clinical characteristics of the two groups are summarised in Table 1.

**Table 1:** Clinical characteristics of patients

Variables	Lymphocyte differential count in BALF		
	>15% (*n* = 24)	≤15% (*n* = 13)	*p* value
Sex (male/female)	21/3	10/3	0.64
Age, years	73 (68–79)	77 (75–78)	0.09
Smoking habit (current/former/never/unknown)	4/15/5/0	0/8/4/1	
RR, /min.	25 ± 8	23 ± 7	0.60
CRP, mg/dl	7.1 ± 5.5	8.0 ± 5.2	0.61
Alb, g/dl	3.2 ± 0.5	3.0 ± 0.4	0.10
LDH, IU/l	323 ± 93	270 ± 63	0.08
Cr, mg/dl	1.09 ± 1.48	0.80 ± 0.22	0.48
KL-6, U/ml	1543 ± 922	1163 ± 874	0.23
SP-D, ng/ml	387 ± 363	215 ± 140	0.11
SP-A, ng/ml	96.4 ± 45.6	84.5 ± 39.0	0.52
pH	7.44 ± 0.03	7.43 ± 0.07	0.63
P/F ratio	261 ± 73	210 ± 89	0.10
Lac, mmol/l	8.7 ± 23.2	4.3 ± 6.9	0.69
CT pattern (UIP/not UIP)	11/13	7/6	0.74
possible UIP pattern	7	1	
Inconsistent with UIP pattern			
Peribronchovascular predominance	5	5	
Upper predominance	1	0	
FVC, % predicted	80.1 ± 15.4	72.0 ± 19.8	0.09
FEV1, % predicted	81.7 ± 17.4	82.1 ± 18.5	0.88
DLco, % predicted	53.9 ± 23.6	54.5 ± 23.8	0.95
Bronchoalveolar lavage			
total volume of retrieved BALF, %	39.3 (33.8–53.7)	43.7 (32.5–52.7)	0.95
Total cell count, /ml	5.0 × 10^5^ (4.0 × 10^5^–5.3 × 10^5^)	3.0 × 10^5^ (2.0 × 10^5^–4.0 × 10^5^)	0.03
Neutrophil, %	15.0 (6.0–22.3)	41.0 (21.0–75.0)	<0.01
Lymphocyte, %	42.7 (33.0–57.5)	7.4 (3.6–9.0)	<0.01
Eosinophil, %	4.5 (2.8–7.4)	2.0 (1.0–4.4)	0.06
Macrophages, %	29.0 (8.3–34.9)	27.0 (10.0–59.0)	0.50
Days from admission until BAL, day	1 (1–2)	2 (1–4)	0.15

Data are presented as median (interquartile range), mean ± SD. *BALF* Bronchoalveolar lavage fluid, *RR* Respiratory rate, *CRP* C-reactive protein, *Alb* Albumin, *LDH* Lactate dehydrogenase, *Cr* creatinine, *KL-6* Sialylated carbohydrate antigen KL-6, *SP-D* Surfactant protein D, *SP-A* Surfactant protein A, *pH* Potential hydrogen, *P/F ratio* PaO_2_/FiO_2_ ratio, *Lac* Lactate, *CT* Computed tomography, *UIP* Usual interstitial pneumonia, *FVC* forced vital capacity, *FEV1* Forced expiratory volume in one second, *DLco* Diffusing capacity for carbon monoxide, *BALF* Bronchoalveolar lavage fluid

### Treatment and outcome

The treatment is summarised in Table 2. Six patients had been treated with immunosuppressants before admission. For AE of CFIIP, all patients were treated with corticosteroids and 33 received corticosteroid pulse therapy (methylprednisolone 1 g/day for 3 days).

**Table 2:** Treatment and outcome of patients

	Lymphocyte differential count in BALF		
Variables	>15% (*n* = 24)	≤15% (*n* = 13)	*P* value
Treatment for CFIIP before admission	5 (21.7%)	5 (38.5%)	0.69
corticosteroid	2 (8.3%)	1 (7.7%)	1.00
corticosteroid + CyA	1 (4.2%)	2 (15.4%)	0.28
pirfenidone	2 (8.3%)	1 (7.7%)	1.00
Treatment for AE of CFIIP			
corticosteroid	10 (41.7%)	3 (23.1%)	0.31
corticosteroid + CyA	6 (25.0%)	4 (30.8%)	0.72
corticosteroid + CY	0 (0.0%)	3 (23.1%)	0.04
corticosteroid + CyA + CY	8 (33.3%)	3 (23.1%)	0.71
Outcome			
Mechanical ventilation	4 (16.7%)	5 (38.5%)	0.23
90-day mortality	4 (16.7%)	8 (61.5%)	<0.01
Overall survival, months	16.5 (5.4–25.8)	1.7 (0.6–4.0)	<0.01
Death during observation period	9 (37.5%)	13 (100.0%)	<0.01

Data are presented as number (percentage). *BALF* Bronchoalveolar lavage fluid, *CFIIP* Chronic fibrosing idiopathic interstitial pneumonia, *AE* Acute exacerbation, *CyA* Cyclosporine A, *CY* cyclophosphamide

Of the nine patients who were mechanically ventilated, five died without withdrawal from the respirator. A total of 22 patients died during the observation period and 12 patients died from AE. Autopsies were performed in two patients who died during their hospitalization. Histopathological examination from these two patients showed the exudative phase of diffuse alveolar damage (DAD) in one patient and the exudative to fibrotic phase of DAD in the other patient.

In the two patients who underwent autopsy, the patient (case 1) with the exudative phase of DAD showed 6.0% lymphocytes and 67% neutrophils in the BALF, and the patient with the exudative to fibrotic phase of DAD (case 2) showed 36% lymphocytes and 17% neutrophils in the BALF. In case 1, the patient received corticosteroid pulse therapy and was mechanically ventilated, but died on the fifth day. In case 2, the patient received corticosteroid pulse and cyclosporine A therapy and was also mechanically ventilated; this patient died on the 40th day.

### Evaluation of the impact of the lymphocyte differential count in BALF on mortality

**Fig. 1:**
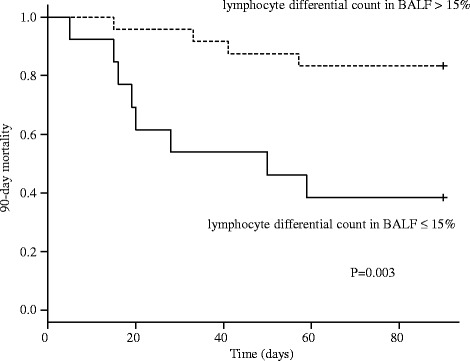
Kaplan-Meier analyses of 90-day mortality of all the patients included in this study stratified by lymphocyte differential count in BALF (lymphocyte differential count in BALF; > 15% vs. ≤ 15%)

**Fig. 2:**
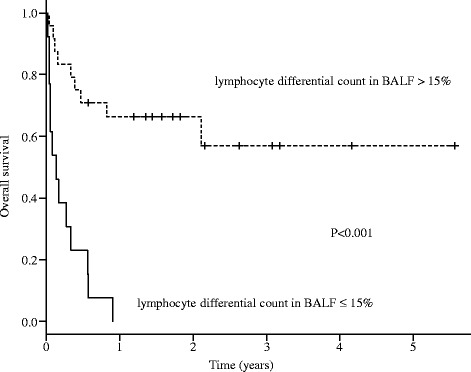
Kaplan-Meier analyses of overall survival of all the patients included in this study stratified by lymphocyte differential count in BALF (lymphocyte differential count in BALF; > 15% vs. ≤ 15%)

The Kaplan-Meier analysis was performed to determine whether the lymphocyte differential count in BALF was associated with the 90-day mortality and OS. The 90-day mortality was significantly higher in the group with a lymphocyte differential count ≤ 15% compared to that of the group with a lymphocyte differential count > 15% (*p* = 0.003; Fig. 1). The OS was also worse in the group with a lymphocyte differential count ≤ 15% compared to that of the group with a lymphocyte differential count > 15% (*p* < 0.001; Fig. 2).

**Fig. 3:**
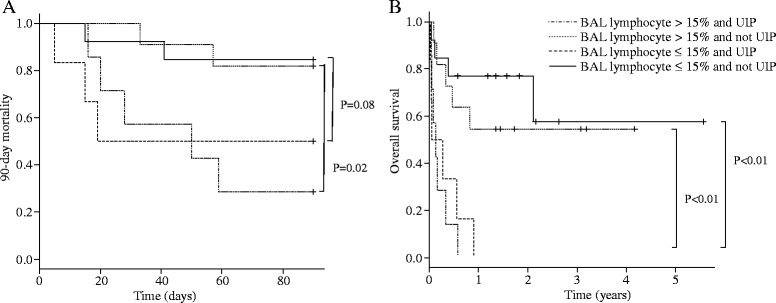
Kaplan-Meier analyzes of 90-day mortality (**a**) and overall survival (**b**) of the patients with both UIP and not UIP stratified by the lymphocyte differential count in BALF (lymphocyte differential count in BALF; > 15% vs. ≤ 15%)

We also evaluated the impact of the lymphocyte differential count in BALF on the mortality of each group with UIP and not UIP, using Kaplan-Meier analysis. Eighteen patients were with UIP, and 19 patients were not UIP. In both groups, the 90-day mortality and OS were significantly worse in the group with a lymphocyte differential count ≤ 15% compared to that of the group with a lymphocyte differential count > 15% (Fig. 3).

### Univariate and multivariate analyses of factors associated with 90-day mortality

The univariate analysis identified three significant favourable prognostic factors: ever smoker, albumin, and BAL > 15% lymphocytes (Table 3). In the multivariate analysis, BAL > 15% lymphocytes was shown to be a statistically significant independent favourable predictor of the 90-day mortality (HR: 0.125; 95% CI: 0.027-0.589; *p* = 0.035).

**Table 3:** Prognostic impacts on the 90-day mortality

Prognostic factors	Univariate analysis			Multivariate analysis		
	HR	95% CI	*P* value	HR	95% CI	*P* value
Age	1.040	0.930–1.170	0.463			
Male	0.409	0.069–2.420	0.325			
Ever smoker	0.190	0.040–0.090	0.037			
Alb	0.210	0.037–1.210	0.081			
KL-6	1.000	0.999–1.000	0.463			
P/F ratio	0.992	0.982–1.000	0.115			
UIP	1.780	0.442–7.180	0.416			
BAL >15% lymphocytes	0.125	0.027–0.589	0.009	0.125	0.027–0.589	0.009
FVC, % predicted	0.956	0.893–1.020	0.199			
DLco, % predicted	1.010	0.974–1.050	0.572			

*Alb* Albumin, *KL-6* Sialylated carbohydrate antigen KL-6, *SP-D* Surfactant protein D, *SP-A* Surfactant protein A, *P/F ratio* PaO2/FiO2 ratio, *UIP* Usual interstitial pneumonia, *BAL* Bronchoalveolar lavage, *FVC* Forced vital capacity, *DLco* Diffusing capacity for carbon monoxide, *HR* Hazard ratio, *95% CI* 95% Confidence interval

## Discussion

In this study, we demonstrated that the lymphocyte differential count > 15% in BALF was associated with favourable outcomes in patients with AE of CFIIP. To the best our knowledge, this is the first study to demonstrate the impact of the lymphocyte differential count > 15% in BALF on the 90-day mortality and OS in patients with AE of CFIIP.

The pathogenesis of AE of CFIIP remains unclear, but the pathological findings of AE of CFIIP were reported as DAD or organizing pneumonia (OP), without other evidence of the organizing phase of DAD [1, 4, 7, 11, 15]. The ATS guidelines indicate that the presence of > 15% lymphocytes in BALF represents a lymphocytic cellular pattern such as OP, nonspecific interstitial pneumonia, etc. [10]. Some of these conditions may respond to corticosteroids. In this study, corticosteroids and other immunosuppressants were more effective in the group with a lymphocyte differential count > 15% than in the group with a lymphocyte differential count ≤ 15%. These results suggest that the 15% lymphocytes in BALF in AE of CFIIP patients is a helpful factor for identifying the therapeutic response and prognosis in these patients. BAL cell differential counts with > 3% neutrophils represent a neutrophilic cellular pattern such as DAD [10] and, in previous reports, patients with DAD were more resistant to corticosteroid and other immunosuppressants and showed a worse prognosis than patients with OP [1, 4, 16, 17]. We analysed association the degree of neutrophil differential count in BALF and OS. The area under the receiver operating characteristic curve for the neutrophil differential count in BALF for predicting the OS in AE of CFIIP was 0.78 [95% CI: 0.59–0.92] (Additional file 1: Figure S1). The univariate analysis identified neutrophil differential count in BALF was associated with OS (HR 1.02, 95% CI 1.01–1.04, *p* < 0.01). In this study, however, 35 patients (94.6%) showed > 3% neutrophils in BALF and a > 3% neutrophil differential count in BALF was not useful in predicting prognosis.

In the group with a lymphocyte differential count ≤ 15%, the OS was significantly inferior to that of the group with a lymphocyte differential count > 15%. Surprisingly, in the group with a lymphocyte differential count ≤ 15%, all five patients that survived the AE died within 1 year from admission. Among these five patients, three died from a recurrence of AE and one died from CFIIP (Table 4). This suggests that in the group with a lymphocyte differential count ≤ 15%, corticosteroid and other immunosuppressants could suppress the appearance of new infiltrations, but could not stop the progression from initial ground-glass opacities to fibrosis. As a result of the progression of fibrosis, respiratory function considerably worsened and AE recurred more frequently. Several previous reports suggested that recombinant human soluble thrombomodulin, pirfenidone, and polymyxin B-immobilized fiber columns may improve survival in AE. The good practice for the management and treatment of AE based on current knowledge, evidence, and available treatment was clinician-led consensus [18]. These treatments may represent additional therapeutic options to improve the prognosis of patients with AE [19–21].

**Table 4:** Cause of death after AE of CFIIP

	Lymphocyte differential count in BALF		
Variables	>15% (*n* = 20)	≤15% (*n* = 5)	*P* value
Reccurence of AE	3 (15.0%)	3 (60.0%)	0.07
CFIIP	1 (5.0%)	1 (20.0%)	0.37
Lung cancer	1 (5.0%)	1 (20.0%)	0.37

Data are presented as number (percentage). *AE* Acute exacerbation, *CFIIP* Chronic fibrosing idiopathic interstitial pneumonia, *BALF* Bronchoalveolar lavage fluid

In the present study, we analysed the prognosis of AE in all CFIIP patients including both UIP and not UIP patients. Usui et al. reported that the computed tomography classification (UIP pattern and not UIP pattern) had no significant effect on survival in AE patients [22], and we considered it valid to place all patients in the CFIIP category. However, although many reports have described the prognosis of AE in IPF patients, few reports have thoroughly investigated the prognosis of AE in not UIP patients [1–3, 7]. Therefore, we classified all patients into the two groups (UIP and not UIP) and analysed the 90-day mortality and OS. Using a cut point of 15% lymphocytes in BALF appeared to be a helpful factor in identifying patients with AE-CFIP who may respond to immunosuppressive therapy, and this appeared to be the case for patients with or without apparent UIP. These findings suggest that >15% lymphocytes in the BALF differential cell count may be a stronger prognostic indicator than whether the underlying histopathologic lesion is UIP or a non-UIP form of CFIIP.

In this study, most of the patients underwent BAL on the first day and received treatment at an early phase. The average total volume of retrieved BALF was 43.3% of the total instilled volume, and this amount was adequate to estimate the BALF cell differential counts [10]. BAL is a safe and well-tolerated procedure [23], and no patient in this study had any serious complications, such as severe worsening of AE. Video-assisted thoracoscopic surgery was too risky to perform during AE. BAL was not only helpful distinguishing AE from infection [10], but it was also useful and safe for evaluating the impact of the lymphocyte differential count in BALF on the mortality of patients with AE of CFIIP.

There are several limitations to the present study. The first is its retrospective design. The second is the fact that it included a relatively small number of patients with AE of CFIIP. The third is that the diagnosis of CFIIP and the classifications were somewhat dependent on the HRCT scanning. Especially, the diagnosis of nonspecific interstitial pneumonia was not adequately accurate because most of the patients were difficult to perform surgical lung biopsies. So, we classified CFIIP into UIP and not UIP and showed “UIP/not UIP” in Table 1. Needless to say, we excluded, in reference to the 2013 ATS guideline, known entities associated with the development of pulmonary fibrosis such as collagen vascular disease and chronic hypersensitivity pneumonitis. The fourth is that we have not compared between BAL in stable CFIIP and in AE of CFIIP. BAL differential counts in stable CFIIP may influence that in AE of CFIIP. However, BAL was performed at the new abnormal infiltration and may represent the acute phase of AE of CFIIP. Finally, because some patients were treated with corticosteroids or other immunosuppressants for CFIIP before AE, the BALF cell differential counts may have been affected. Prospective studies with larger patient cohorts are required to overcome these limitations.

## Conclusions

In conclusion, our findings indicate that the lymphocyte differential count > 15% in BALF may be associated with good outcomes in patients with AE of CFIIP. Further studies are needed to confirm our findings, to elucidate the mechanism of AE of CFIIP, and to develop the optimal treatment strategies for AE of CFIIP.

## Additional file

Additional file 1: Figure S1. The receiver operating characteristic curve for the neutrophil differential count in BALF for predicting the OS in AE of CFIIP. (PPTX 78 kb)

## Abbreviation

95% CI: 95% confidence interval
AE: Acute exacerbation
ATS/ERS/JRS/ALAT: American Thoracic Society/European Respiratory Society/Japanese Respiratory Society/Latin American Thoracic Association
BALF: Bronchoalveolar lavage fluid
CFIIP: Chronic fibrosing idiopathic interstitial pneumonia
DAD: Diffuse alveolar damage
HR: Hazard ratio
IPF: Idiopathic pulmonary fibrosis
OP: Organizing pneumonia
OS: Overall survival
UIP: Usual interstitial pneumonia
