# Supplementary material for: Impact of lymphocyte differential count > 15% in BALF on the mortality of patients with acute exacerbation of chronic fibrosing idiopathic interstitial pneumonia
Source: BMC Pulm Med. 2017 Apr 20;17:67. doi: 10.1186/s12890-017-0412-8 (PMC5397815; doi:10.1186/s12890-017-0412-8)
Supplement: Additional file 1: Figure S1. — The receiver operating characteristic curve for the neutrophil differential count in BALF for predicting the OS in AE of CFIIP. (PPTX 78 kb) [file 12890_2017_412_MOESM1_ESM.pptx]

## Slide 1
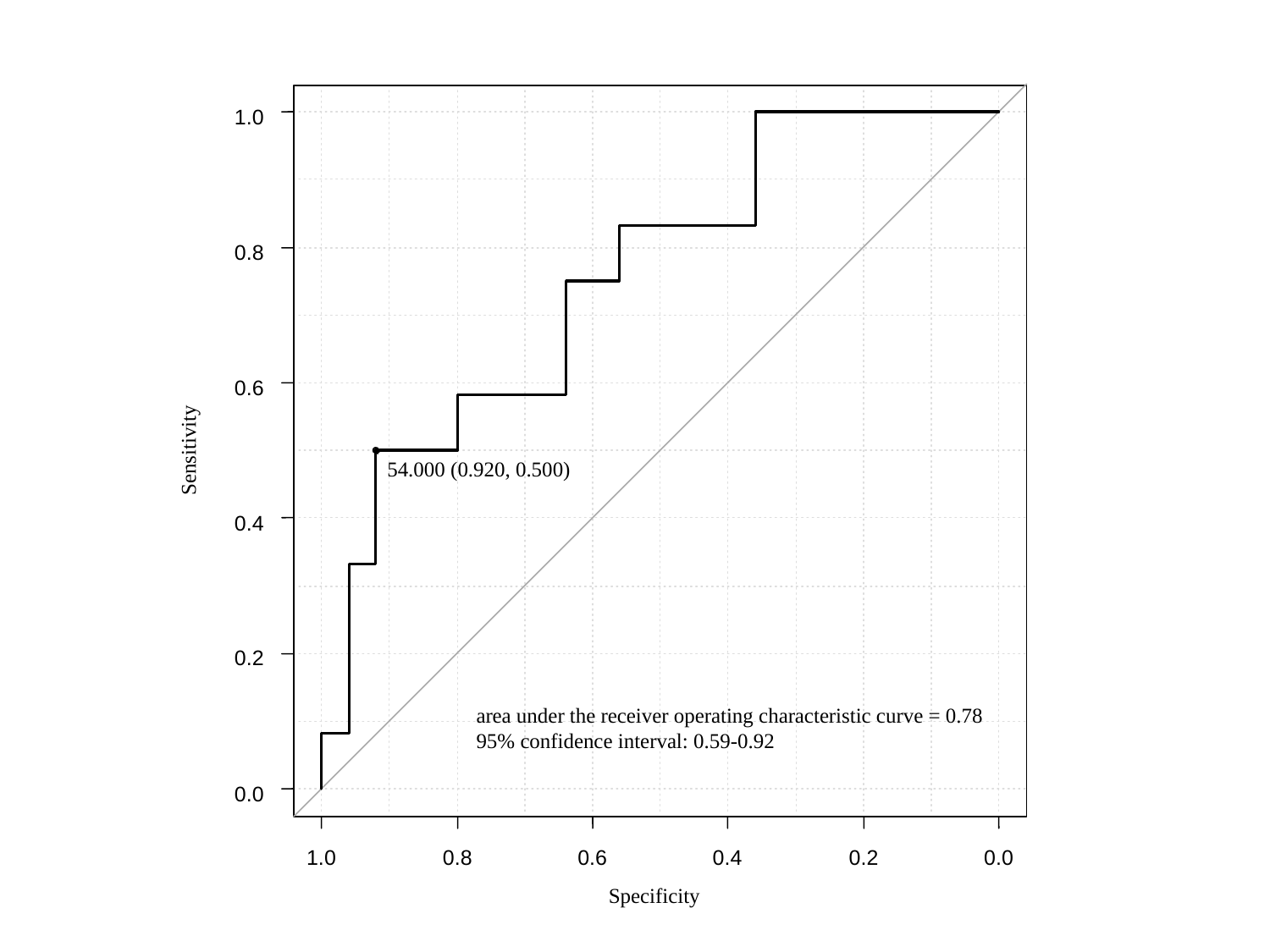

1.0
0.8
0.6
Sensitivity
54.000 (0.920, 0.500)
0.4
0.2
0.0
1.0
0.8
0.6
0.4
0.2
0.0
Specificity
area under the receiver operating characteristic curve = 0.78
95% confidence interval: 0.59-0.92
